# Cost‐Effective Solid‐State NMR Of Fungal Glucans: A Case Study On *Schizosaccharomyces pombe*

**DOI:** 10.1002/chem.71081

**Published:** 2026-05-02

**Authors:** Ananya Singh, Teresa Massam‐Wu, Mohan Balasubramanian, Wing Ying Chow

**Affiliations:** ^1^ Department of Physics University of Warwick Coventry UK; ^2^ Warwick Medical School University of Warwick Coventry UK

**Keywords:** ^13^C‐labeling, β‐1,3‐glucan, fungal cell wall, quantification, thermal response

## Abstract

Solid‐state NMR provides molecular‐level insights into fungal cell walls, but full ^13^C‐enrichment is costly and limits biological replication. Here, we show that 10% ^13^C‐glucose labeling in *Schizosaccharomyces pombe* is sufficient to resolve the major carbohydrate resonances and quantify glucan composition with high reproducibility. We then examined cell wall composition at two temperatures, both in whole cells and in extracted wall fractions. Interestingly, whole cell spectra indicated minor, nonsignificant changes with temperature, while extracted walls revealed differences between the rigid glucan matrices with β‐1,3‐glucan especially enriched at 36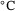. Together, these results establish low‐level ^13^C labeling as a cost‐effective strategy for fungal solid‐state NMR and highlight the complementary strengths of whole cell and extracted wall‐level analysis in uncovering how the effect of elevated temperature is encoded in cell wall architecture.

## Graphical abstract

A cost‐effective solid‐state NMR strategy enables replication and semi‐quantitative assessment of inherent variation of the fungal cell wall. Whole cell and cell wall extractions yield subtly different compositional differences, revealing molecular adaptations invisible to microscopy.

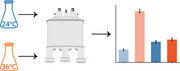

## 1 Introduction

Solid‐state nuclear magnetic resonance (NMR) spectroscopy offers a powerful and non‐destructive means to probe the molecular architecture of fungal cell walls in situ [1, 2]. Yet, a major limitation is the high cost of isotopically labeled substrates, especially for full ^13^C‐enrichment, which can constrain experimental scale and statistical reproducibility. This challenge is particularly relevant when studying fungal cell walls, where structural heterogeneity and environmental influences result in biological variation that demands replications to ensure reproducibility. ^13^C NMR have been employed to resolve polysaccharide composition and organization in fungi using natural abundance and isotopically enriched (labeled) samples. Fernando et al. [3] analyzed unlabeled cells from *Aspergillus fumigatus* and *Candida albicans* using dynamic nuclear polarization, revealing a conserved carbohydrate core and rigid α‐glucan‐chitin domains. Kang et al. [4] employed solid‐state NMR to investigate the cell wall architecture of uniformly labeled *A. fumigatus*, demonstrating that chitin andα‐1,3‐glucan forms a hydrophobic scaffold surrounded by a hydrated matrix of β‐glucan and glycoproteins. Building on these studies, we apply a complementary approach based on fractional ^13^C labeling in *Schizosaccharomyces pombe* (*S. pombe*), enabling cost‐effective, reproducible analysis of glucan remodeling under elevated temperature.

Yeasts are widely utilized as model systems in basic and applied biosciences, including biotechnology, life sciences, and healthcare [5]. Among them, the fission yeast *S. pombe* stands out as a robust and versatile model for eukaryotic biology. It is highly conducive to genetic modifications, cost‐effective for experimental studies, and shares significant similarities with human genetic machinery governing the cell cycle [6, 7, 8, 9]. The *S. pombe* cell wall consists of a compact glucan matrix composed mainly of β‐1,3‐glucan (46%–54%), with contributions from α‐1,3‐glucan (18%–28%) and other minor components [10]. These polysaccharides govern the cell shape, mechanical strength, and adaptive responses to stressful environmental conditions [11, 12, 13]. Analyzing the composition and adaptive remodeling of glucans is crucial for understanding fungal responses to environmental stresses. This knowledge has significant implications for their survival, ecological roles, and resilience to climate change [14], and is also vital for formulating targeted antifungal strategies [15, 16].

In *S. pombe*, glucan biosynthesis and fungal cell wall remodeling have been investigated using biochemical assays, enzymatic digestions [11, 17, 18], solution‐state NMR [10, 19, 20], and most recently, solid‐state NMR, which was used to probe GH13‐domain enzyme‐mediated remodeling of insoluble α‐glucans [21]. Thus far, most studies focus on whole cells or soluble fractions. Analyses of extracted cell walls can complement these approaches by enabling a more detailed characterization of the insoluble polysaccharide matrix, allowing precise quantification of glucan composition, identification of rigid structural domains, and capture molecular‐level adaptations that may be obscured in bulk whole cell measurements. We propose that a more comprehensive view of fungal cell wall architecture and its dynamic remodeling under different conditions may be obtained by combining whole cell and wall‐specific analyses.

Temperature is a key environmental factor influencing fungal physiology, affecting not only growth rates but also cell wall structure and enzyme activity [20]. Prior studies have shown that temperature shifts can alter glucan profiles in various fungi, affecting the balance between α‐ and β‐glucan types [12]. However, while temperature‐dependent effects on cell wall biosynthesis have been explored in yeast and filamentous fungi [22, 23], a detailed molecular‐level characterization of how *S. pombe* specifically remodels its cell wall in response to elevated temperature, particularly distinguishing changes in the wall itself from the whole cell variations, has not been reported. Here, we extract cell walls using mechanical disruption with a bead beater, without applying acid or alkali treatments, thereby preserving native polysaccharide structures while removing interfering intracellular components. This approach enables precise analysis of glucan composition and its remodeling under elevated temperature, with broad relevance for industrial fermentation [24], pathogenic fungal stress responses [25], and environmental adaptation [26].

Given the complexity of fungal cell wall systems and the cost of isotopic enrichment, our primary goal in this study is to develop a cost‐effective and scalable solid‐state NMR strategy for quantifying glucan composition using only fractional 10% ^13^C‐labeling. To assess the feasibility of reduced isotope enrichment, we compared whole cells grown with 10% and 100% uniformly ^13^C‐labeled glucose at 24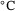 and 36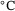. One‐dimensional ^13^C spectra under both labeling conditions enabled relative quantification of α‐ and β‐1,3‐glucan signals. Quantitative analysis using ssNake [27] peak fitting showed that 10% labeling faithfully reproduces glucan trends seen in fully labeled samples. Despite lower signal to noise ratio in 2D ^13^C–^13^C spectra, key linkages remain identifiable. Compared to uniform ^13^C labeling, fractional labeling thus allows reduction in the use of ^13^C glucose even with biological replication, allowing quantification of biological variation, leading to more reproducible results.

To distinguish cell wall‐specific changes from broader cellular responses, we further combined this labeling strategy with the extraction of the insoluble wall matrix, to enable direct comparison between intact whole cells and their extracted cell walls, allowing statistically robust analysis of localized remodeling.

Our results reveal a temperature‐dependent shift in the cell wall composition, marked by enrichment of β‐1,3‐glucan and two subtypes of α‐1,3‐glucans: Aa (structural, reducing‐end) and Ab (likely branched) in the extracted wall matrix. These localized changes were not evident in whole cell analyses, underscoring the potential of using extracted cell wall preparations to detect adaptive remodeling that reinforces the wall under heat stress. Broadly, this study presents a robust, cost‐effective solid‐state NMR framework for investigating fungal wall dynamics, with relevance for microbial physiology, biotechnology and environmental science.

## 2 Results and Discussion

**FIGURE 1:**
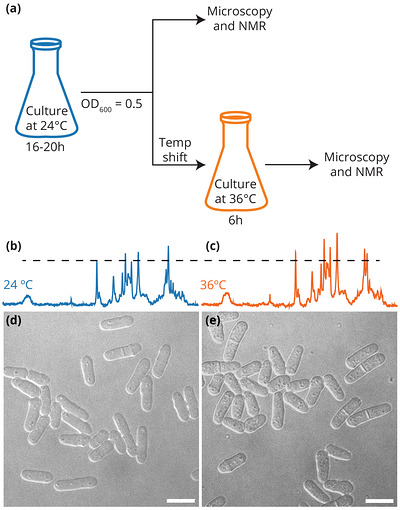
Overview of the experimental design and basic characterization. (a) Experimental workflow under two temperature conditions: standard (24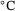) and elevated (36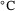). 1D ^13^C CP NMR spectra of cells grown at (b) 24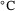 (OD_600_ = 0.5), and (c) 36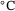 (6h from the temperature shift). The NMR spectra share a common vertical scale. (d,e) Microscopic images of *S. pombe* cells under the two temperature conditions showing that their morphology is not affected. Scale bar: 10μm.

To understand how *S. pombe* adapts its cell wall architecture in response to temperature, cultures were initially grown at 24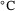 until reaching an optimal optical density (OD_600_) of 0.5, after which they were shifted to 36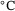 to induce an elevated temperature condition (Figure 1). We combined phenotypic observations with solid‐state NMR using ^13^C‐labeled glucose. Morphological changes were assessed by microscopy, while quantitative analysis of polysaccharide composition was performed by solid‐state NMR on samples obtained from culture at both 24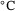 (standard) and 36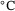 (elevated temperature) conditions.

Light microscopy revealed no significant morphological differences between *S. pombe* cells grown at 24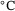 and 36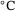 (Figure 1d–e). Both populations maintained similar rod‐like shape and size, indicating that the elevated temperature does not induce major alterations in cell morphology. In contrast, solid‐state NMR spectra showed differences between the two temperature conditions (Figure 1b,c), revealing molecular‐level changes in cell wall composition that are not apparent from microscopy alone. Growth analysis revealed striking biosynthetic differences: cultures grown at 36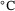 reached significantly higher cell densities than those at 24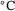 (Figure S1). The biomass yield at 36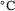 was approximately threefold greater than 24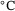, suggesting that cells grown at elevated temperature exhibit faster proliferation and potentially enhanced metabolic flux [28]. These findings motivated a comparative molecular analysis of cells grown at the two temperatures using solid‐state NMR to probe temperature‐dependent changes in cellular composition.

In this study, we focus on the rigid components of the fungal cell wall using solid‐state NMR, and employed cross‐polarization (CP) experiments to selectively enhance signals from immobile polysaccharides under standard (24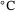) and elevated temperature (36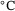) conditions. CP is particularly effective for detecting signals from rigid polysaccharides such as β‐glucans and α‐glucans, enabling relative comparison of the rigid glucan fractions across the two temperatures. The resulting analysis is semi‐quantitative, as the CP intensities depend on nearby ^1^H spins and molecular mobility. Direct polarization (DP) spectra provided a valuable qualitative overview of spectral complexity and signal overlap in fungal samples (Figure S4), but were not used for quantitative comparison due to sensitivity differences relative to CP measurements.

**FIGURE 2:**
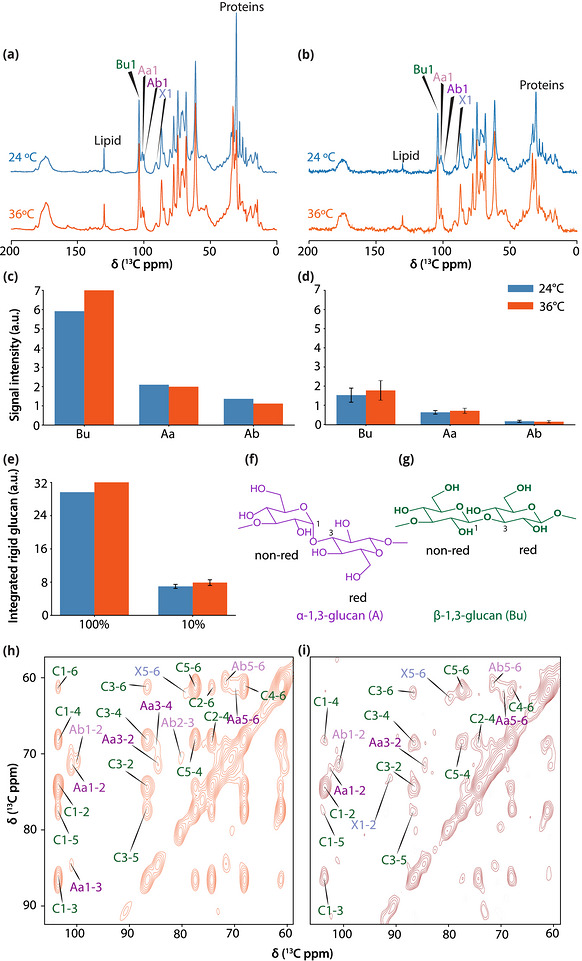
Comparison of 100% and 10% uniformly ^13^C‐labeled *S. pombe* cells cultured at two different temperatures: 24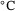 (blue, top) and 36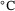 (orange, bottom). ^13^C CP NMR spectra of (a) 100% and (b) 10% uniformly ^13^C‐labeled cells (1024 scans, 600 MHz, 10 kHz MAS rate). C1 peaks of glucans are indicated, while full assignments are provided in the Supporting Information (Table S2). Glucan abbreviations: β‐1,3‐glucan (Bu), α‐1,3‐glucan type A (Aa), α‐1,3‐glucan type B (Ab), and an unknown glucan (X). Bar plots showing relative CP‐derived signal intensities of β‐1,3‐glucan (Bu), α‐1,3‐glucan type A (Aa), and α‐1,3‐glucan type B (Ab) derived from (c) 100% and (d) 10% labeling schemes, respectively. (e) Integrated rigid glucan content estimated from integration of ^13^C CP spectra, showing comparable trends across labeling levels, with only minor, non‐significant temperature‐dependent variations in whole cell samples. Error bars represent the standard error of the mean (SEM) from three biological replicates for 10% labeled samples; no error bars are shown for 100 % labeled samples due to *n* = 1. Statistical significance (*p* ≤ 0.05) was assessed using a two‐sided Student's *t*‐test. (f,g) Chemical structures of the identified glucan types, indicating their reducing (red), and non‐reducing (non‐red) rings. (h,i) 2D ^13^C–^13^C DARR spectra of 100% (16 scans) and 10% (128 scans) labeled cells grown at 36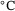 (mixing time: 20ms, 600 MHz, 10 kHz MAS rate).

We first evaluated whether reduced ^13^C‐labeling could still yield reliable carbohydrate structural information. We compared whole *S. pombe* cells grown in media containing 100% or 10% uniformly ^13^C‐labeled glucose at the two different temperatures. CP‐based solid‐state NMR spectra revealed qualitatively similar 1D profiles for both labeling conditions (Figure 2a,b). In both cases, we observed signals at consistent chemical shift values, corresponding to key carbohydrate motifs, in particular the following characteristic C1 signals: β‐1,3‐glucan (∼103.6 ppm); two subtypes of α‐1,3‐glucan, including a reducing end‐type A (∼101 ppm) and a structurally distinct type B (∼100 ppm); and an unknown glucan, X (∼90.7 ppm). Despite the expected reduction in sensitivity, 2D ^13^C–^13^C dipolar‐assisted rotational resonance (DARR) spectra from the 10% labeled samples retained characteristic signals of β‐1,3‐glucan such as C1–C3 and C1–C4 correlations (Figure 2h,i). All key signals were observed at 20 ms DARR mixing time and no additional crosspeaks were observed at longer mixing times (Figure S2). While some weaker correlations, especially for X, were not fully resolved, the majority of diagnostic signals were observed, supporting the use of fractional labeling for reliable identification of major glucan structures.

To estimate the relative abundance of rigid glucan components based on the most resolved C1 signals, we compared three processing methods: manual integration and deconvolution in TopSpin, and Lorentzian peak fitting in ssNake [27]. All three methods produced consistent trends, but ssNake was chosen for its superior baseline correction, more accurate resolution of overlapping signals, and higher reproducibility. These features are critical for the complex spectra of heterogeneous whole cell samples.

The integrated CP signal corresponding to rigid glucans, obtained from ssNake‐based deconvulation was markedly higher in fully ^13^C‐labeled cells compared to 10% labeled cells at both temperatures (Figure 2e). At 24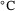, the total signal in 100% labeled cells reached about 30 a.u., whereas 10% labeled cells showed a significantly lower mean integrated area of around 7 ± 5 a.u. A similar trend of approximately four‐fold difference was also observed at 36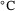. This reflects the expected reduction in sensitivity due to lower isotopic enrichment in the 10% samples, as well as fewer ^13^C–^13^C dipolar couplings contributing to signal build‐up under CP conditions. Despite differences in total spectral area, the relative distribution and temperature‐dependent trends of individual rigid glucans were consistent across labeling conditions (Figure 2c,d). β‐1,3‐glucan was the dominant component and increased with temperature in both fully and fractionally labeled cells. This increase suggests a temperature‐dependent reinforcement of the β‐glucan backbone, likely contributing to enhanced wall rigidity at elevated temperatures. α‐1,3‐glucan (Aa) remained relatively stable, with a slight increase in 10% labeled cells and a minor decrease in 100% labeled ones, suggesting minimal fluctuation within experimental variability. In contrast, α‐1,3‐glucan (Ab) showed a consistent decrease of about 15% at higher temperature in both datasets, possibly reflecting temperature‐sensitive modulation of branching or packing interactions involving this subtype of α‐1,3‐glucan. Importantly, these trends were captured reproducibly in both labeling contexts, indicating that fractional labeling preserves not only the overall composition but also subtle temperature‐dependent variations in individual glucan types.

Moreover, using 10% ^13^C‐labeling may help mitigate metabolic perturbations associated with fully labeled carbon sources. Previous studies have shown that high ^13^C enrichment can alter central metabolic fluxes by affecting enzyme kinetics and regulatory feedback, which could unintentionally shift the balance of polysaccharide biosynthesis [29]. In this context, the relatively stable compositional profiles observed in 10% labeled cells suggest that fractional labeling can better preserve the physiological state of the cell, supporting its use as a biologically relevant and cost‐effective strategy for solid‐state NMR studies of glucan architecture.

**FIGURE 3:**
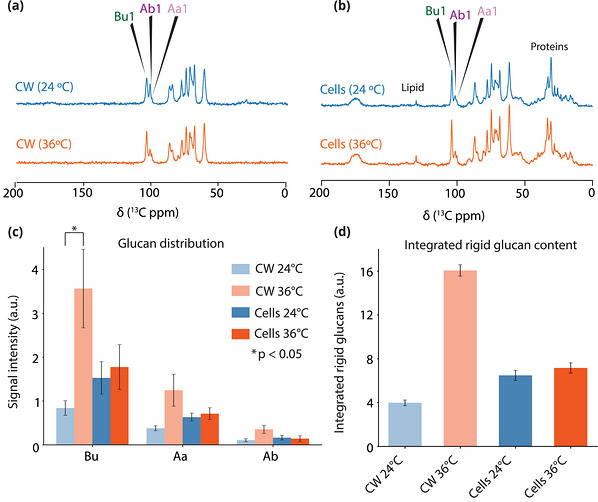
Comparison of 10% uniformly ^13^C‐labeled *S. pombe* extracted cell wall preparation and whole cell samples at two different temperatures: 24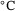 (blue) and 36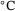 (orange). ^13^C CP NMR spectra of 10% uniformly ^13^C‐labeled (a) extracted cell walls and (b) whole cells, respectively (1024 scans, 600 MHz, 10 kHz MAS rate). Glucan C1 signals are labeled. Glucan abbreviations: β‐1,3‐glucan (Bu), α‐1,3‐glucan type A (Aa) and α‐1,3‐glucan type B (Ab). (c) Bar plot showing relative CP‐derived signal intensities of β‐1,3‐glucan (Bu), α‐1,3‐glucan type A (Aa) and α‐1,3‐glucan type B (Ab) derived from (a) and (b). (d) Integrated rigid glucan content estimated from integrations of the whole ^13^C range of CP spectra. Error bars represent the standard error of the mean (SEM) calculated from biological replicates (*n* = 3). Statistical significance was assessed using a paired two‐sided Student's *t*‐test to identify differences between temperatures (*p ≤ 0.05). Elevated temperature results in a significant increase in β‐1,3‐glucan content in extracted cell wall samples (*p* = 0.026), while no significant changes are observed for α‐1,3‐glucan in either whole cells or extracted cell walls (*p* >0.05).

Having established that 10% ^13^C‐labeled whole cells can yield reproducible relative glucan profiles, we next compared spectra obtained from whole cells and extracted cell wall preparations to investigate the subcellular distribution and temperature‐dependent remodeling of glucans. We investigated several methods of physically breaking up the cells, including glass beads and cryomilling, settling finally on a bead beater method that maximized sample yield while eliminating protein signals (Figure 3a,b). At 24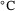, the integrated CP signal assigned to rigid glucans in whole cells was nearly 1.75‐fold higher than that in extracted cell walls (Figure 3d), suggesting that a portion of rigid, wall‐associated glucans may be disrupted, solubilized, or lost during wash steps and processing of the cell wall preparation. In contrast, at 36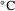, the extracted cell walls contained nearly twice as much glucan as whole cells, indicating increased retention of rigid glucans within the insoluble matrix at elevated temperature. This temperature‐dependent redistribution implies enhanced polymerization and incorporation of glucans into the covalently cross‐linked wall, likely reinforcing mechanical strength under elevated temperature.

Glucan subtype analysis (Figure 3c) revealed β‐1,3‐glucan (Bu) as the dominant component across all conditions. In the extracted cell walls, Bu signal increased four‐fold from 24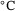 to 36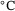, compared to a modest 16% rise in whole cells, supporting temperature‐driven densification of the β‐1,3‐glucan network via enhanced polymerization or packing. The structurally anchoring α‐1,3‐glucan (Aa) similarly rose by 70% in extracted walls but only ∼11% in whole cells, indicating increased retention or incorporation into the insoluble matrix at higher temperatures. Meanwhile, α‐1,3‐glucan (Ab) declined slightly in whole cells but tripled in the extracted walls, suggesting temperature‐induced stiffening or crosslinking that promotes its integration into the rigid scaffold. Paired, two‐sided *t*‐tests showed that elevated temperature significantly increased the CP‐derived β‐1,3‐glucan content in the extracted cell wall samples (*p* = 0.026), while no significant changes were observed for αα‐1,3‐glucans in either whole cells or extracted cell walls (*p* >0.05). Together, these trends suggest that *S. pombe* undergoes coordinated glucan remodeling in response to elevated temperature, increasing both the quantity and insolubility of wall‐associated β‐ and α‐1,3‐glucans. This adaptation likely contributes to the thermal resilience of the cell wall by reinforcing its architecture with a denser, more cross‐linked glucan network.

To complement the compositional analysis of rigid components, we performed 1D insensitive nuclei enhanced by polarization transfer (INEPT), 2D INEPT–Total through bond correlation spectroscopy (INEPT‐TOBSY) [30], and direct polarization (DP) experiments to probe mobile regions in both whole cells and extracted cell walls of *S. pombe*. Compared with CP, the INEPT‐based spectra revealed a different set of sugar signals, reflecting the enhanced detection of dynamic polysaccharides such as α‐galactomannan and β‐1,6‐glucan (Figure S3). Clear spectral differences were observed between 24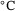 and 36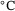, with the samples produced under the elevated temperature condition yielding sharper, better‐resolved, and more intense INEPT–TOBSY cross‐peaks, consistent with increased molecular mobility (Figure S7). Comparison of 10% versus 100% ^13^C‐labeled samples showed that key dynamic sugar correlations were retained under fractional labeling, despite reduced overall intensity (Figure S4). Finally, whole cell samples consistently produced stronger INEPT signals than cell wall extracts at both temperatures (Figure S3), indicating the likely additional contributions of dynamic intracellular metabolites in intact cells.

## 3 Conclusion

This study establishes a cost‐effective and reliable strategy for probing microbial, in particular fungal cell walls, using solid‐state NMR spectroscopy. By comparing 10% and 100% ^13^C‐glucose labeling in *S. pombe*, we show that low‐level enrichment, combined with spectral fitting of CP‐based measurements, provides reproducible semi‐quantitative comparisons of rigid carbohydrates and allows multiple biological replications, especially relevant for whole cell samples.

In our temperature shift model, a statistically significant difference was observed between whole cell and extracted cell wall samples obtained at 24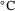, highlighting that extracted cell wall preparations are particularly informative for detecting subtle biochemical adaptations that can complement whole cell measurements. A drawback of cell wall extractions is that a larger volume of fungal cell culture is required as a result of material loss during the extraction process. Fractional labeling can therefore also make it more economical to study extracted cell wall preparations, where detailed information on relative changes in rigid polysaccharide components is required.

Overall, this work demonstrates that low‐level ^13^C‐labeling enables robust, reproducible semi‐quantitative analysis of rigid fungal cell wall glucans. Whole cell measurements provide a global view of glucan composition, while extracted cell walls offer detailed insights into localized structural changes. Combining these approaches enable comprehensive studies of glucan remodeling under elevated temperature, with direct consequences for understanding fungal adaptation under the global context of rising temperature and environmental change.

## Conflicts of Interest

The authors declare no conflict of interest.

## Supporting information

The authors have cited additional references within the Supporting Information [31, 32, 33, 34, 35]. Moreover, details of experimental and statistical analysis are provided.

## Data Availability

The data that support the findings of this study are openly available in FigShare at https://dx.doi.org/10.6084/m9.figshare.32114128 and in the Warwick Research Archive Portal (WRAP) at https://wrap.warwick.ac.uk/196299/.
