# Supplementary material for: Cost‐Effective Solid‐State NMR Of Fungal Glucans: A Case Study On Schizosaccharomyces pombe
Source: Chemistry. 2026 May 2;32(27):e71081. doi: 10.1002/chem.71081 (PMC13380364; doi:10.1002/chem.71081)
Supplement: Supplementary file 1 — The authors have cited additional references within the Supporting Information [31, 32, 33, 34, 35]. Moreover, details of experimental and statistical analysis are provided. [file CHEM-32-e71081-s001.pdf]

Supplementary Information

Cost-Effective Solid-state NMR of Fungal Glucans: A case study on *Schizosaccharomyces pombe*

Ananya Singh,[1] Teresa Massam-Wu,[2] Mohan Balasubramanian,[2] Wing Ying Chow[1*]

[1] Department of Physics, University of Warwick, Coventry, UK, CV4 7AL.

[2] Warwick Medical School, University of Warwick, UK, CV4 7AL.

*Corresponding author: w.ying.chow@warwick.ac.uk

Table of Contents

Supplementary Information 1

Availability of the raw data 3

Experimental details 4

Growth curves at the two temperatures (Figure S1 & Table S1) 6

DARR of 10% ^{13}C-labelled samples at different mixing times (Figure S2) 7

INEPT spectra of 10% ^{13}C-labelled cells and cell walls (Figure S3) 8

CP, INEPT, and DP spectra of 10 and 100% ^{13}C-labelled cells (Figure S4) 9

Spectral fitting (Figure S5) 10

DARR of 100% ^{13}C-labelled samples (Figure S6) 11

INEPT-TOBSY of fractional ^{13}C-labelled sample (Figure S7) 12

^{13}C chemical shifts of carbohydrates of *Schizosaccharomyces pombe* (Table S2) 13

References 14

Availability of the raw data

The data that support the findings of this study are openly available on FigShare at https://doi.org/10.6084/m9.figshare.32114128 and in the Warwick Research Archive Portal (WRAP) at https://wrap.warwick.ac.uk/196299/.

Experimental details

Preparation of ^{13}C-labelled cell samples

Schizosaccharomyces pombe wild-type (MBY192) was streaked from glycerol stock onto yeast extract agar (YEA) plates and grown at 30 °C. For isotope labelling, cells were cultured in 500 mL of YEA liquid medium containing 2.5 g yeast extract, 22.5 mL of 0.5% adenine, 15 g ^{12}C glucose, and 1.5 g uniformly ^{13}C-labeled glucose (Cambridge Isotope Laboratories). Cultures were grown in 2 L Erlenmeyer flasks at 25 °C with 200 rpm shaking (Innova 42 Incubator Shaker Series) to an optical density (OD_{600}) of 0.5-0.7, and then either harvested or shifted to 36 °C for 6 h to induce a thermal response before harvesting. Cells were pelleted by centrifugation at 1500g for 10 min (Eppendorf Centrifuge 5810R), washed three times with sterile water, and stored at −20 °C before packing them in the solid-state NMR rotor.

Cell Wall Extraction

Frozen cell pellets were thawed and resuspended in sterile water, using a vortex mixer (Vortex-Genie 2 Scientific Industries) as needed to ensure homogeneous suspension. Cell disruption was performed using a bead beater (MP FastPrep-24) with 0.5 mm acid-washed glass beads, applying 15 cycles of 60 s at 4 m/s. Lysis efficiency was confirmed microscopically. The lysate was separated from the glass beads by decanting the supernatant, followed by centrifugation at 4500g for 10 min (Eppendorf Centrifuge 5424) to isolate the crude cell wall pellet. The pellet was washed three times with 5% NaCl to remove electrostatically bound intracellular proteins, and then four times with sterile water. Final pellets were centrifuged at 4500g (Eppendorf Centrifuge 5424), the supernatant discarded, and samples stored at −20 °C before rotor packing for solid-state NMR studies.

Solid-state NMR experiments

Solid-state NMR experiments were performed using Bruker AVANCE NEO spectrometers at the Department of Physics, University of Warwick, operating at ^{1}H Larmor frequencies of 600 MHz (14.1 T) and 602 MHz (14.15 T), respectively. All experiments were conducted at a set temperatures of 268 K under 10 kHz MAS using a 3.2 mm EFree probe. ^{13}C chemical shifts were externally referenced to the carbonyl peak of alanine at 177.8 ppm (relative to tetramethylsilane/TMS at 0 ppm). Typical radiofrequency field strengths were 90-100 kHz for ^{1}H decoupling, 70-100 kHz for ^{1}H hard pulses, and 55-70 kHz for ^{13}C pulses.

For NMR measurements, samples were packed into 3.2 mm MAS rotors with comparable amounts of material (~ 30 mg) to ensure consistent filling factors across experiments. Packing was performed by gently transferring the hydrated sample into the rotor followed by centrifugation to achieve uniform and reproducible packing density. Care was taken to

avoid drying during preparation. All samples were measured in their hydrated, native state, and rotor caps were sealed immediately after packing to maintain sample hydration throughout the experiments.

A combination of 1D and 2D ssNMR experiments was employed to analyse both rigid and mobile components. Rigid domains were probed using ^{13}C cross-polarisation (CP) with a recycle delay of 2 s, while mobile components were detected using a J-coupling-based refocused INEPT experiment, again with a recycle delay of 2 s. Direct polarisation (DP) experiments were additionally performed with a recycle delay of 15 s. All 1D experiments (CP, INEPT, and DP) were acquired with 1024 scans unless otherwise stated. Dipolar-based 2D ^{13}C-^{13}C DARR correlation spectra (mixing time of 20 ms) enabled resonance assignment of rigid polysaccharides through intramolecular cross-peaks and were acquired with 128 scans for 100% ^{13}C-labelled samples and 16 scans for 10% labelled samples. Mobile molecules were further characterised by 2D INEPT-TOBSY spectra acquired with 64 scans. Chemical shift assignments were validated by comparison with entries in the Complex Carbohydrate Magnetic Resonance Database (CCMRD)[1]. Protein signals were also observed in whole cell samples. The observed ^{13}C chemical shifts for carbohydrates are summarised in Table S2.

Statistical analysis

Molecular composition was quantified from the CP ^{13}C spectra by calculating the relative intensities of resolved C1 signals corresponding to α- and β-glucans. Lorentzian line-shape fitting of these C1 peaks was performed using ssNake[2] (v1.6.1) with 2000 evaluations, and the integral value of each fitted C1 peak was used to represent the intensity of the corresponding glucan. Mean values and standard deviations were calculated from three independent samples. The standard error of the mean (SEM) was obtained by dividing the standard deviation by the square root of the number of replicates. To assess variability across samples, deviations (d) from the mean were squared (d^2), and the sum of squared deviations was used to determine the overall variance. A two-sided Student's t-test[3] was additionally performed in Python (v3.13.9) using the SciPy library (v1.16.3) to evaluate the statistical significance of the differences between conditions. This approach was applied across all conditions (extracted cell wall and whole cell samples at 24 °C and 36 °C). Total glucan levels (in arbitrary units) were estimated by summing the integrated values of resolved C1 peaks corresponding to α- and β-glucans from the CP spectra. Final bar plots were generated using Python, incorporating the NumPy and Matplotlib modules.

Growth curves at the two temperatures

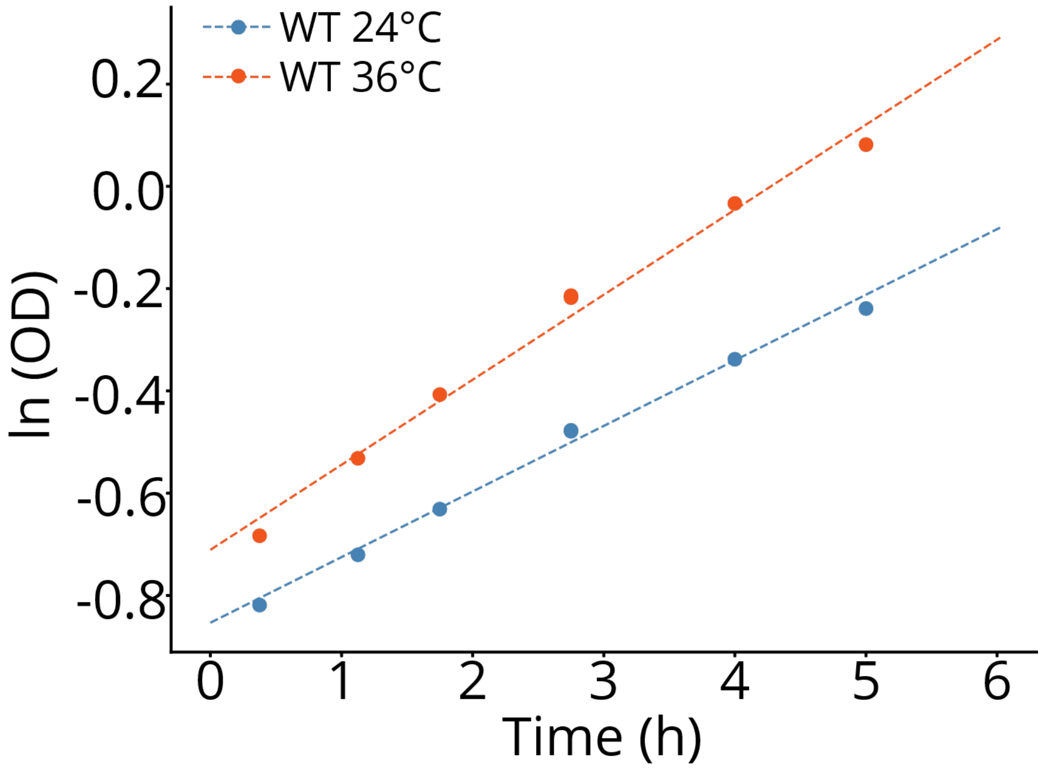

Figure S1. Growth curves of *S. pombe* cells at 24 °C and 36 °C, plotted as ln(OD) versus time. Linear regression fits of the exponential phase are shown with the corresponding growth rates (μ), doubling times (td), and coefficients of determination (R^2) as tabulated.

**Temperature**	**μ (h^{-1})**	**td (h)**	**R^2**
24 °C	0.128	5.42	0.99
36 °C	0.167	4.15	0.99

Table S1. Linear regression fits of the exponential phase are shown with the corresponding growth rates (μ), doubling times (td), and coefficients of determination (R^2) as tabulated.

DARR of 10% ^{13}C-labelled samples at different mixing times

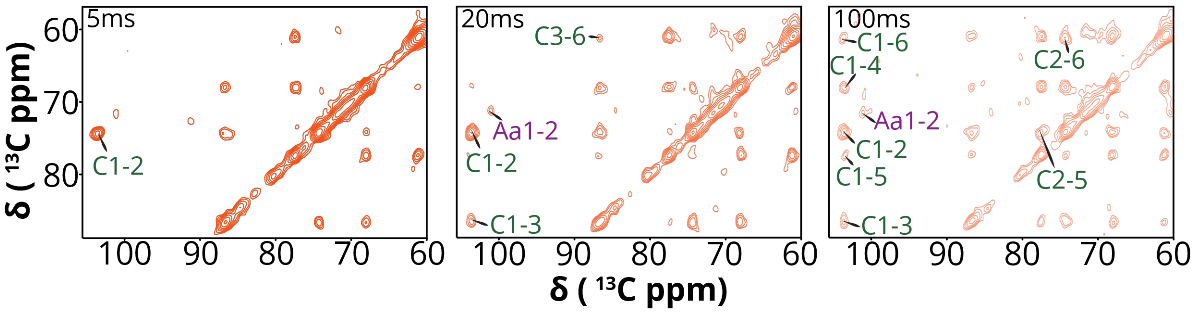

Figure S2. 2D ^{13}C-^{13}C DARR spectra of 10% ^{13}C-labelled *S. pombe* WT cells at 36 °C, acquired with mixing times of 5 ms (left), 20 ms (middle), and 100 ms (right) to probe different correlation distances. Experiments were performed on a 600 MHz spectrometer with 10 kHz MAS.

INEPT spectra of 10% ^{13}C-labelled cells and cell walls

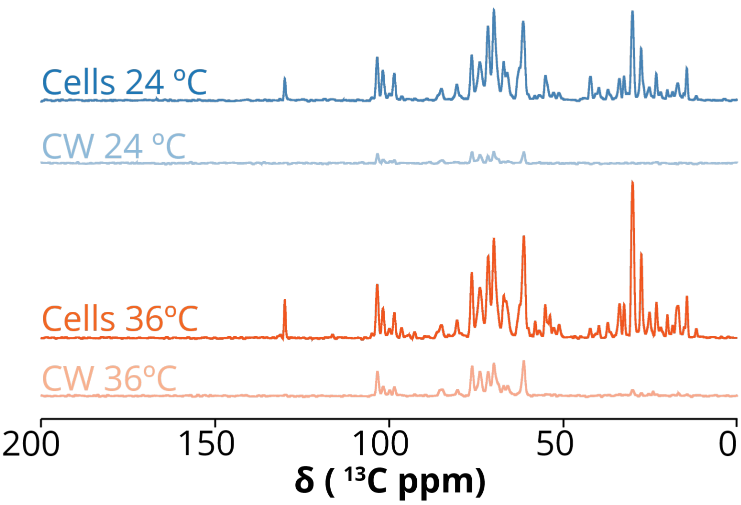

Figure S3. 1D ^{13}C INEPT spectra of 10% ^{13}C-labelled *S. pombe* WT whole cells (dark) and extracted cell walls (light) at 24 °C (blue) and 36 °C (orange), highlighting signals from mobile molecular components. Spectra were acquired on a 600 MHz spectrometer under 10 kHz MAS with 1024 scans.

CP, INEPT, and DP spectra of 10 and 100% ^{13}C-labelled cells

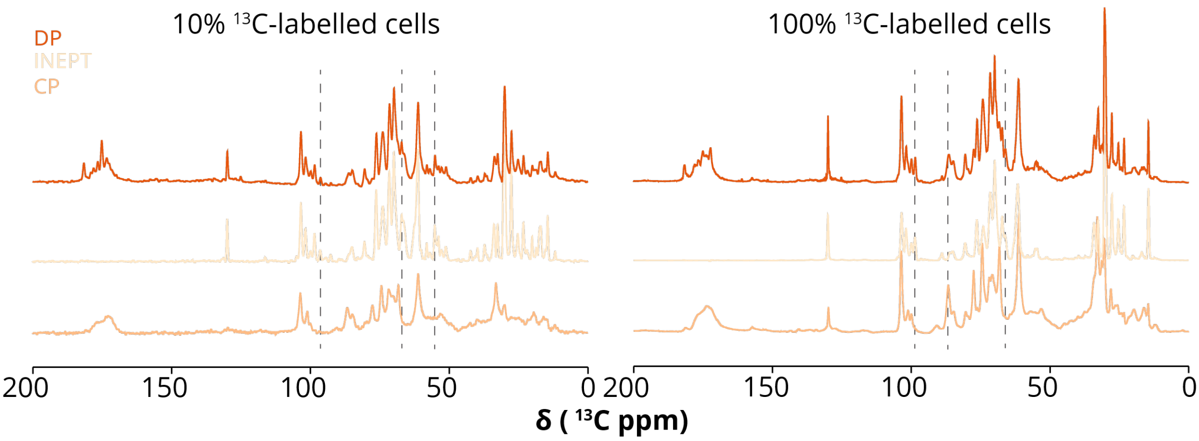

Figure S4. Comparison of 1D CP, INEPT, and DP ^{13}C spectra of *S. pombe* whole cells with 10% (left) and 100% (right) ^{13}C labelling. All spectra were acquired on a 600 MHz spectrometer under 10 kHz MAS with 1024 scans. CP spectra (2 ms contact time) emphasise rigid components, INEPT highlights mobile molecular regions, and DP spectra (15 ms recycle delay) detect all ^{13}C nuclei regardless of mobility.

Spectral fitting

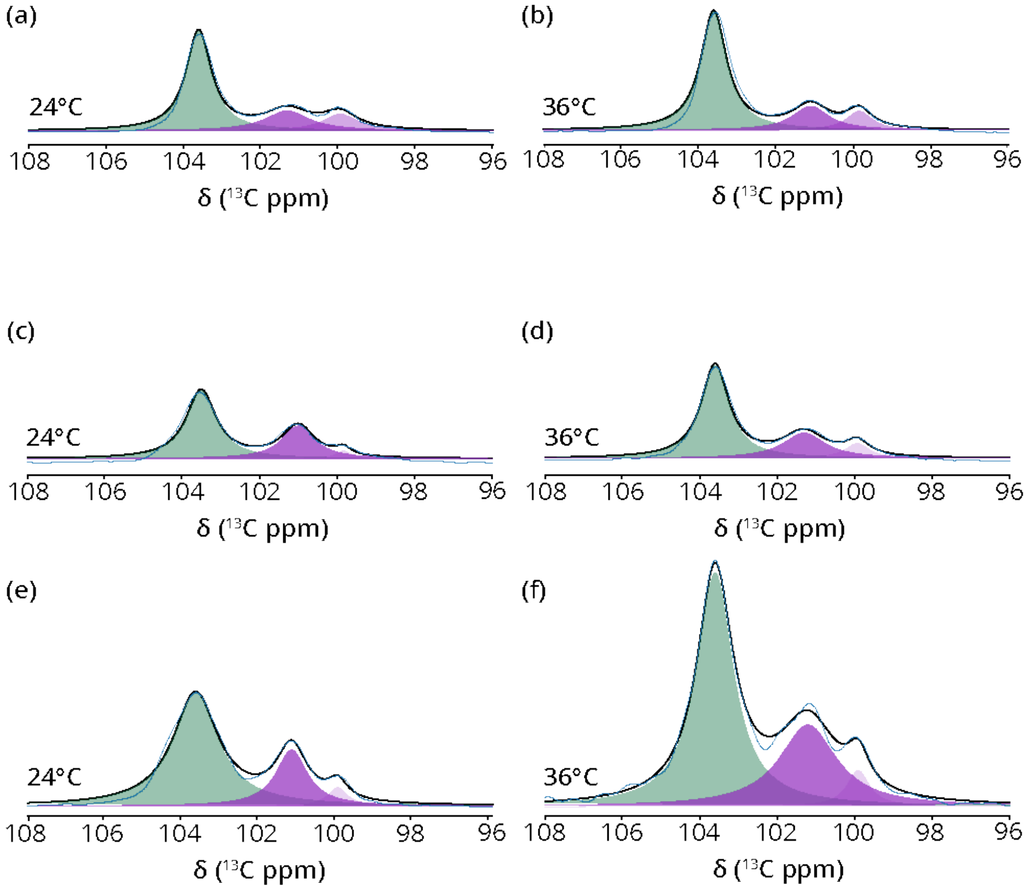

Figure S5. Spectral fitting of the most resolved C1 peaks of β-1,3-glucan and α-1,3-glucans using ssNake. Panels (a) and (b) correspond to 100% ^{13}C-labelled cells, panels (c) and (d) to 10% ^{13}C-labelled cells, and panels (e) and (f) to extracted cell walls from 10% ^{13}C-labelled cells.

DARR of 100% ^{13}C-labelled samples

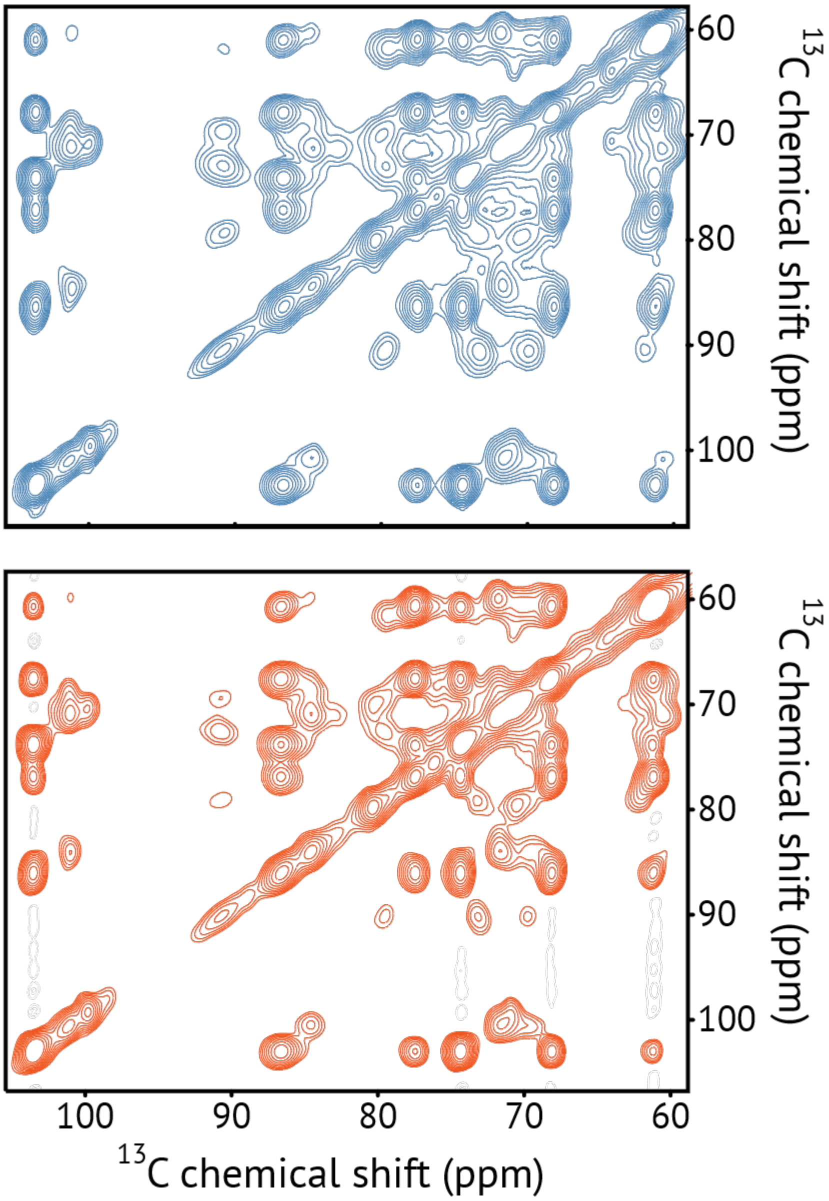

Figure S6. 2D ^{13}C-^{13}C DARR spectra of 100% ^{13}C-labelled *S. pombe* WT cells at 24 °C (top) and 36 °C (bottom), acquired with mixing time 20 ms. Experiments were performed on a 600 MHz spectrometer with 10 kHz MAS at 268 K.

INEPT-TOBSY of fractional ^{13}C-labelled sample

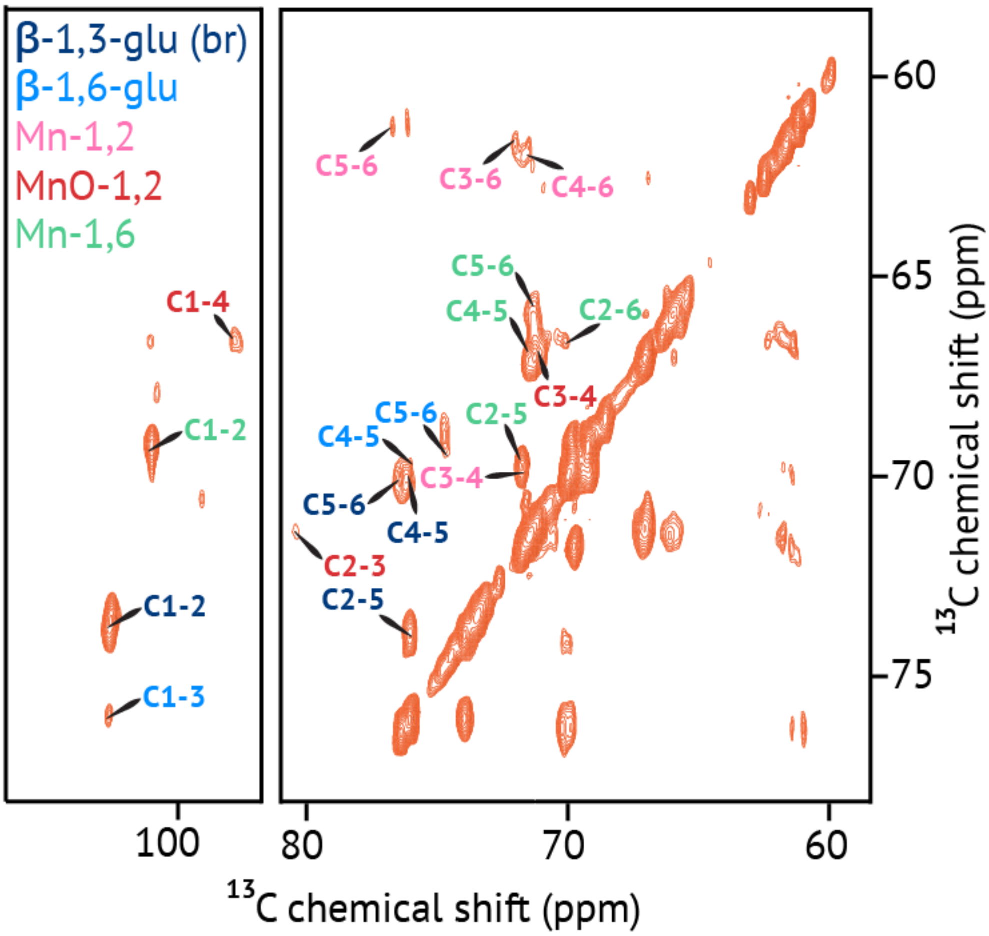

Figure S7. 2D ^{13}C-^{13}C INEPT-TOBSY spectra of 10% ^{13}C-labelled *S. pombe* WT cells at 36 °C (exp time 11 hours). Experiments were performed on a 1 GHz spectrometer with 10 kHz MAS at 268 K.

^{13}C chemical shifts of carbohydrates of *Schizosaccharomyces pombe*

**Carbohydrate**	**C1**	**C2**	**C3**	**C4**	**C5**	**C6**	**Experiment**	**References**
α-1,3-glucan (a)	101	71.4	84.6	68	70.1	60.8	^{13}C-^{13}C DARR	Bhanja *et al.* 2014[4]
α-1,3-glucan (b)	99.9	70.7	79.9	73.1	71.8	60.5	^{13}C-^{13}C DARR	Jacob *et al.* 2025[5]
β-1,3-glucan (unbr)	103.5	74.3	86.6	68.1	77.4	61.2	^{13}C-^{13}C DARR	Shim *et al.* 2007[6] Fairweather *et al.* 2004[7]
X	90.7	73.1	69.9	70	79.6	62.2	^{13}C-^{13}C DARR	
β-1,3-glucan (br)	103	73.5		69.5	76	70.1	^{13}C-^{13}C INEPT-TOBSY	
β-1,6-glucan (H)	103.4	73.8	76.1	70.1	74.7	69.0	^{13}C-^{13}C INEPT-TOBSY	
Mn-1,2			71.7	70.2	76.4	61.2	^{13}C-^{13}C INEPT-TOBSY	Jacob *et al.* 2025[5]
MnO-1,2	98.6	80.4	71.4	67			^{13}C-^{13}C INEPT-TOBSY	
Mn-1,6	101.7	70.2	74.4	67.6	71.3	66.2	^{13}C-^{13}C INEPT-TOBSY	

Table S2. ^{13}C chemical shifts of carbohydrates in *S. pombe* cell walls. “a” and “b” denote different allomorphs. Unidentified (–). Branched (Br). Reducing end (O). Unbranched (unbr). Rigid sugar resonances in ^{13}C–^{13}C DARR align with literature, while deviations in mobile sugars from ^{13}C–^{13}C INEPT–TOBSY are mainly at C4 and C5 of Mn-1,2; C4 of MnO-1,2; and C1, C2, C3, and C5 of Mn-1,6.

References

1. X. Kang, W. Zhao, M. C. Dickwella Widanage, A. Kirui, U. Ozdenvar, T. Wang, J. Biomol. NMR 2020, 74, 239.
2. S. G. J. van Meerten, W. M. J. Franssen, A. P. M. Kentgens, J. Magn. Reson. 2019, 301, 56.
3. Student, Biometrika 1908, 6, 1.
4. S. K. Bhanja, D. Rout, P. Patra, I. K. Sen, C. K. Nandan, S. S. Islam, Bioact. Carbohydr. Diet. Fibre 2014, 3, 52.
5. Jacob, A. H. Willet, M. G. Igarashi, M. El Hariri El Nokab, L. A. Turner, A. K. A. Alsanad, T. Wang, K. L. Gould, Proc. Natl. Acad. Sci. U.S.A. 2025, 122, e2505509122
6. J.-H. Shim, K.-J. Sung, M.-C. Cho, W.-A. Choi, Y. Yang, J.-S. Lim, D.-Y. Yoon, J. Microbiol. Biotechnol. 2007, 17, 1513.
7. J. K. Fairweather, Glycobiology 2004, 14, 775.
